# Stability Control of a Biped Robot on a Dynamic Platform Based on Hybrid Reinforcement Learning

**DOI:** 10.3390/s20164468

**Published:** 2020-08-10

**Authors:** Ao Xi, Chao Chen

**Affiliations:** Laboratory of Motion Generation and Analysis, Faculty of Engineering, Monash University, Clayton, VIC 3800, Australia; ao.xi@monash.edu

**Keywords:** biped robot, reinforcement learning, stability control, Gaussian processes, DQN (*λ*)

## Abstract

In this work, we introduced a novel hybrid reinforcement learning scheme to balance a biped robot (NAO) on an oscillating platform, where the rotation of the platform is considered as the external disturbance to the robot. The platform had two degrees of freedom in rotation, pitch and roll. The state space comprised the position of center of pressure, and joint angles and joint velocities of two legs. The action space consisted of the joint angles of ankles, knees, and hips. By adding the inverse kinematics techniques, the dimension of action space was significantly reduced. Then, a model-based system estimator was employed during the offline training procedure to estimate the dynamics model of the system by using novel hierarchical Gaussian processes, and to provide initial control inputs, after which the reduced action space of each joint was obtained by minimizing the cost of reaching the desired stable state. Finally, a model-free optimizer based on DQN (*λ*) was introduced to fine tune the initial control inputs, where the optimal control inputs were obtained for each joint at any state. The proposed reinforcement learning not only successfully avoided the distribution mismatch problem, but also improved the sample efficiency. Simulation results showed that the proposed hybrid reinforcement learning mechanism enabled the NAO robot to balance on an oscillating platform with different frequencies and magnitudes. Both control performance and robustness were guaranteed during the experiments.

## 1. Introduction

Humanoid biped robots have high degrees of freedom that allow them to imitate human motions with the purpose of completing complex tasks under different working environments [1]. Biped robots are also required to be able to adapt to irregular terrains and maintain full functionality when completing tasks, leading to a high requirement of controllers to guarantee the robustness under disturbances. Biped robots normally contain more than twenty degrees of freedom, which leads to a highly sophisticated control strategy to deal with their kinematic complexity to maintain balance [2]. Thus, stability control of humanoid biped robots has always been a difficult task for researchers in the field of robotics and artificial intelligence. The goal of all these studies in these fields is to propose new controllers to provide higher robustness, stability performance, and adaption to more complex terrains [3].

Stability control strategies of biped robots can be divided into conventional control methods and artificial intelligence-based control methods. Conventional control methods are based on the dynamic model of the robot, which involves the position of center of mass of each joint and each link, the length and width of each link, etc. These methods generate control signals for joints according to the deviation between the desired zero moment point (ZMP) and the measured ZMP [4,5,6,7,8]. The problem of conventional control methods is that they highly rely on the accuracy of the dynamics model. Biped robots normally contain uncertainties in joint friction, contact force with the ground, and other unmeasurable variables. Therefore, it is extremely difficult to obtain an accurate model that contains both the robot dynamics and the environment disturbances. In addition, traditional techniques to obtain the dynamic model of the system are computationally demanding and time consuming. On the other hand, conventional controllers are designed for a specific robot model with particular parameters for that robot. For example, a novel conventional control strategy is originally proposed for an NAO robot [9]. Although it works perfectly on NAOs, it is not applicable to Michigan’s Cassie-series robots as the parameters of these robots are completely different [10]. The designed controller is able to work in different robots only if the parameters of these robots are identical or at least highly similar to one another. Thus, attempting to generalize these controllers to enable most biped robots to be adaptable and flexible to different terrains still remains difficult for researchers. 

Machine learning (ML) algorithms have been proposed recently to overcome the above-mentioned limitations, since they do not require the dynamics model of the robot but still achieve the same control performance [11]. Although the integration between ML and robotics is still at their early stage, the huge potentials of ML have been proved to have a promising future in robotic control applications. Valdez, in 2019 [12], proposed an integrated machine learning structure that consists of fuzzy logic control (FLC) and conventional control techniques, which was successfully applied and validated on a real robot to complete the speed control tasks. Juang proposed a fully connected recurrent neural network (RNN) optimization technique to generate a multi-objective gait and applied it to control the gait of a real NAO robot [13]. Ferreira compared the support vector regression (SVR) and neural fuzzy (NF) network based on ZMP error correction to balance the robot [14]. Sun utilized a neural network (NN) to approximate the unknown model of the robot, where it was able to deal with system uncertainties during the task of balancing and posture control [15]. Saputra proposed a biologically inspired RNN to control the biped robot, where the neurons of the network were used to represent the muscular and sensor system of the body [16]. Although these NN structure-based approaches have been proven to be able to replace the classic control methods, and provide an efficient and robust control performance, the performance of these NNs are limited by their teacher controllers, which means if the teacher controller contains errors or the training data are not sufficient enough, the performance of the network will be significantly affected. Compared with approaches utilizing NN to estimate the system model, reinforcement learning (RL) techniques, however, directly employ interactions between the agent (robot) and the environment, where the agent can complete the desired tasks by gathering experience directly from its environment without being trained by target controllers.

Several studies, for example Rodic and co-workers [17], have been conducted involving RL as part of the control scheme to control the biped robot. They proposed a novel hybrid control strategy by combining the model-based dynamic controller with RL feedback controller. Guerreo [18] proposed a supervised RL approach that combined supervised learning (NN) with RL. Experimental results showed that the algorithm was able to be applied on a physical robot to complete the tasks of docking and grasping. However, none of these methods applied RL as the direct joint level controllers, the actions were simply some discrete predefined action samples. The main limitation of their works is that only discrete states and actions were considered, where large errors or oscillations were introduced to the controller. Many recent attempts have been made by Hwang [19,20] with the purpose of applying Q-learning as the joint controller to maintain the balance of the robot of uneven terrains as well as the imitation of human postures. Although continuous actions were generated using Gaussian distribution in their work, the RL structure still relies on a look-up table, meaning that the convergence time would be extremely long. Wu [21] however, based on Gaussian basis function, employed an abstraction method to reduce the number of state cells needed and applied it on a biped robot to maintain balance on a rotating platform. Simulation results showed that it was able to achieve high precision control with a reduced dimension of state space. One of the major drawbacks to adopting abstraction is that with the generation of the clustering, the actions for each cluster may differ significantly. To achieve better control performance, actions between two nearby clusters (or states) must be transferred smoothly. Seo utilized deep Q-learning (DQN) and the inverted pendulum model (IPM) to control the robot joints to complete the push recovery, where DQN was able to deal with a huge number of states without generating a look-up table or clusters [22]. Shi, in 2020 [23], employed the deep deterministic policy gradient (DDPG) to improve the precision of attitude tracking control of a biped robot. They proposed an offline pre-training scheme to provide the prior knowledge to the online training in the real physical environment. Garcia, in 2020 [24], proposed a safe RL algorithm for improving the walking pattern of a NAO robot to allow it to walk faster than the pre-defined configuration. The results showed that their approach was able to increase the learning speed while still reducing the number of falls of the robot during the process of learning. The above methods are all considered as model-free reinforcement learning (MFRL). Although steady state performance can be guaranteed after convergence as no model-induced error is introduced to the system, one major challenge of MFRL is that the convergence time (learning time) is extremely long since no model is used to provide prior knowledge of the system.

Model-based reinforcement learning (MBRL), as an alternative approach, is able to reduce the learning time by generating a transition model within the algorithm [10]. The transition model is essentially a mapping f(s,a)→ s ′ from the state s of the system at a given time step, and the applied action a (or policy) to the state  s ′ at the next time step. This model is also known as the stochastic model, where it is defined by a probability distribution over the future state [25]. The most commonly used techniques are Gaussian processes. Deisenroth proposed probabilistic inference for learning control (PILCO) to estimate the transition model of the system, where it only requires very little training data [26]. Policies are consequently improved using the analytical results of the policy gradient. Experimental results show that the algorithm is able to control the physical robotics systems, where the learning procedure is facilitated from scratch in only a few trials [27,28]. Other techniques such as expectation maximization (EM) [29], or dynamic Bayesian networks (DBN) [30], have been proven to successfully estimate the system models. Compared with MFRL, MBRL only requires a small amount of training data, leading to a short convergence time, but it highly relies on the accuracy of the estimated model. Since the policy is computed based on the transition model, any change of model, induced by measurement error or lack of information for estimation, may lead to an exponential change of the resulting policy. Efforts must be made to update the model for each iteration to increase the model accuracy, which is extremely computationally expensive compared to updating the policy directly.

As a result, many attempts aiming at combining model-based approaches with model-free learning techniques have been made in recent years. Nagabandi in 2018 proposed a hybrid RL structure that combined NN dynamics models with model predictive control (MPC) to achieve excellent sample complexity in a model-based RL algorithm. Then, they further proposed a NN dynamics model to initialize a model-free learner, where the algorithm guarantees the sample efficiency as well as the control performance [31]. Pong instead combined MFRL with MBRL by introducing the temporal difference models (TDMs) [32]. The proposed RL structure combined the benefits of model-free and model-based RL, where it can be trained with model-free learning and used for model-based control tasks. Feinberg, in 2018, proposed a model-based (MB) value expansion method that incorporated the predictive system dynamics model into model-free (MF) function estimation to reduce sample complexity. According to their algorithm, the predicted dynamics were fitted first, then a trajectory was sampled from the replay buffer to update the actor network, after which an imagined future transition was obtained and was used to update the critic network. Both of the MB and MF parts were used to update the actor and critic network during the learning process [33]. Gu incorporated a particular type of learned model into the proposed Q-learning scheme with normalized advantage functions to improve sample efficiency. They used a mixture of planned iterative LQG and on-policy trajectories, and then generated additional synthetic on-policy rollouts using the learned model from each state visited along the real-world rollouts. At the same time, the model itself was being refit for every n episodes to obtain an effective and data-efficient model learning algorithm [34]. Hafez employed a novel curious meta-controller, where it alternated adaptively between model-based and model-free control. In the end, both the MB planer and MF learner were improved mutually. The method improved the sample efficiency and achieved near-optimal performance [35]. However, these methods were only validated on simple fully-observed models (the walker or the swimmer) under ideal conditions, where no disturbances were added to the environment. They also required a huge amount of computations as both MBRL and MFRL were updated within the iteration during the online learning procedure. In addition, none of the above approaches consider the motor limitations, mechanical constraints, or disturbances. Thus, they are not applicable for physical biped robot joint control tasks to maintain balance as it requires precise joint velocity control at any time step to achieve the minimum steady state error under a dynamic environment. 

As a conclusion, first of all, for the model-based RL to achieve a better control performance, the estimated model must be precise enough to contain all information of the system. The problem of it is that it is highly computationally demanding and time consuming to obtain such precise models. Secondly, for the model-free RL, the convergence time is extremely long as there is no prior knowledge of the system for the pure model-free RL. Thirdly, the distribution mismatch problem always exists in other types of HRL frameworks [33,34,35,36]; therefore, to overcome this problem further calculations are required to determine which policy is better, leading to less efficiency of these HRL frameworks. Fourthly, the recent HRL frameworks only validated on simple fully-observed robotic models in ideal environments; thus, they are not suitable for complex biped models such as NAO robots. Thus, a hybrid RL structure has the potential to overcome stability control challenges for biped robots, which has not been fully explored.

To guarantee the fast convergence of MFRL and to achieve the sample efficiency of MBRL, a novel hybrid reinforcement learning framework (HRL) incorporating the inverse kinematics (IK) is introduced in this work, where it successfully establishes a serial connection between MBRL and MFRL. This framework is applied it to a complex biped model (NAO robot) to maintain sagittal and frontal balance on a dynamic platform with disturbances. We combined the hierarchical Gaussian processes (HGP) as the model-based estimator with DQN(λ) as the model-free fine tuning optimizer, where HGP is utilized to predict the dynamics of the system and DQN(λ) is utilized to find the optimal actions. The benefit of using this framework is that the model-based estimator does not need to obtain a very precise dynamic model of the system, which significantly reduces the number of training data as well as the computational load. Secondly, this hybrid structure successfully avoids the distribution mismatch problem (mentioned in ref. [33,34,35,36]) while combining model-based RL with model-free RL. Thirdly, as the estimated model is obtained, and the action space is significantly reduced; therefore, the convergence time of the model-free optimizer is consequently reduced. As a conclusion, the efficiency of the proposed HRL is improved compared with pure model-based RL, pure model-free RL, and other types of HRL approaches. Simulation results show that the proposed algorithm was able to be applied on a NAO robot to maintain balance on a dynamic platform under different oscillations at all times. The adaptability was also guaranteed for the robot on a platform with different rotating frequencies and magnitudes.

This paper is organized as follows: Section 2 provides the model of the NAO robot and the corresponding platform. Several assumptions and constraints are elaborated in detail. The state space and action space of the system are defined accordingly. In Section 3, the overview of RL is presented, as well as the basic principle of GP and the corresponding optimization techniques. Then, we illustrate the structure of the proposed hierarchical Gaussian processes (HGP) and briefly explain the function of each layer, after which we explain how the reduced action space is defined for each state according to the immediate cost function. Finally, we present the MFRL to improve the policy based on the temporal difference learning method. Section 4 is the experiment setup as well as all simulation results. In Section 5 is the conclusion of this work and the discussion about the potential future extension of the proposed HRL.

## 2. Formulation of the Problem

**Figure 1:**
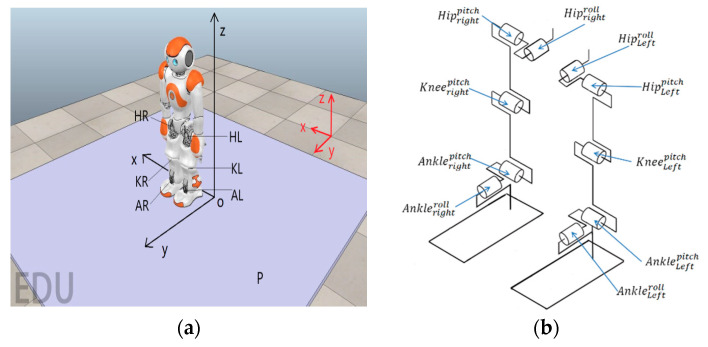
(**a**) The overview of NAO robot and the platform. (**b**) The simplified mechanism of the proposed NAO robot.

As shown in Figure 1, a NAO robot stands on a platform, where the platform is rotating along the x-axis and y-axis at the same time. The platform is employed here to simulate the uneven surface of the real environment, where oscillations are introduced to the system to imitate real external disturbances. Compared with the flat surface under the experimental environment, such a platform is able to provide a more complex environment to make the robot learn a more robust and efficient controller. This paper will conduct experiments to test not only the efficiency of learning procedures (including the convergence time, number of training data, etc.), but also the robustness of the learned controller to deal with complex environments.

At the beginning of all experiments, NAO is initialized to the default standing posture. The control task of the upper body, including the torso, arms, hands, and head, is ignored as it can be considered as a point mass using the inverted pendulum model (IPM) [37]. Both of the robot’s foot soles are always attached to the platform and will not slip at any times. The ankle joint has two degree of freedoms (DoFs) which can rotate along the  x-axis (roll) and y-axis (pitch). The knee joint has one DoF that allows it to rotate along the x-axis. The hip joint has two DoFs that are pitch and roll, which allows it to rotate along the x-axis and y-axis, respectively.

As can be seen from Figure 1, the robot is at its initial position, where the red and black frames are the world frame and the robot frame, respectively. P is the rotating platform which can rotate along the x-axis (roll) and y-axis (pitch) in the world frame. $H_{R}$ and $H_{L}$ represent the right hip and the left hip, respectively. $K_{R}\left(K_{L}\right)$ and $A_{R}\left(A_{L}\right)$ indicate the knee joint and the ankle joint, respectively. When the robot is at its default standing posture, the distance between the hip and the ankle is $l_{A_{L}}^{H_{L}}=l_{A_{R}}^{H_{R}}=0.2019\;m$, the distance between two hip joints are $l_{H_{R}}^{H_{L}}=0.1\;m$, and the length of thigh and tibia are $l_{K_{R}}^{H_{R}}=l_{K_{L}}^{H_{L}}=0.1\;m$ $l_{A_{R}}^{K_{R}}=l_{A_{L}}^{K_{L}}=0.1029\;m$, respectively. The distance between the two hip joints is assumed to be fixed as $l_{H_{R}}^{H_{L}}=0.1\;m$ at all times. There are 10 control inputs applied at hips (four control inputs), knees (two control inputs), and ankles (four control in puts). For hip joints, the control inputs are the change of pitch angles ($\Delta q_{H_{R}}^{p},\;\Delta q_{H_{L}}^{p}$), and the change of roll angles ($\Delta q_{H_{R}}^{r},\;\Delta q_{H_{L}}^{r}$), respectively. For knee joints, the control inputs are the change of pitch angle ($\Delta q_{K_{R}}^{p},\;\Delta q_{K_{L}}^{p}$). For the ankle joints, the control inputs are the change of pitch angles ($\Delta q_{A_{R}}^{p},\;\Delta q_{A_{L}}^{p}$), and the change of roll angles ($\Delta q_{A_{R}}^{r},\;\Delta q_{A_{L}}^{r}$), respectively. Thus the control inputs, or in our case, the action applied on NAO at any time step is a 10 dimensional array that can be written as $A={\left[\Delta q_{A_{R}}^{p},\;\Delta q_{A_{L}}^{p},\Delta q_{A_{R}}^{r},\;\Delta q_{A_{L}}^{r},\Delta q_{K_{R}}^{p},\;\Delta q_{K_{L}}^{p},\Delta q_{H_{R}}^{p},\;\Delta q_{H_{L}}^{p},\Delta q_{H_{R}}^{r},\;\Delta q_{H_{L}}^{r}\right]}^{T}$. The action space for each element is defined as Δq∈[−0.1, 0.1]. Assuming that each dimension of the action space contains 10 discrete actions, the total action space has $10^{10}$ possible combinations. If the dimension of action space can be reduced, the computation load for RL can be reduced significantly, leading to a reduced training complexity for MB system estimator and an increased learning speed for the MF optimizer. Thus, we employed the most commonly used and the most straightforward approach in robotics, i.e., the inverse kinematics (IK) technique, to reduce the dimension of applied action a by defining the mechanical constraints. The roll action and pitch action of the left ankle are equal to the right ankle at all times ($\Delta q_{A_{R}}^{r}=\Delta q_{A_{L}}^{r},\;\Delta q_{A_{R}}^{p},=\Delta q_{A_{L}}^{p}$); the left knee always executes the same action as the right knee ($\Delta q_{K_{R}}^{p},=\Delta q_{K_{L}}^{p}$); the change of pitch angle of the left and right hip joints are identical for any time step ($\Delta q_{H_{R}}^{p},=\Delta q_{H_{L}}^{p}$); the roll angle of both hip joints are fixed to default and will not change during the entire experiment ($\Delta q_{H_{R}}^{r},=\Delta q_{H_{L}}^{r}=0$). As a result, the action space is reduced to $A={\left[\Delta q_{A}^{p},\;\Delta q_{A}^{r},\Delta q_{K,\;}\Delta q_{H}^{p}\right]}^{T}$. Since the length of the thigh and tibia are fixed at all times, we can further reduce the dimension of the action space by applying the inverse kinematics (IK) constraints. Given the pitch and roll action of the ankle joints ($\Delta q_{A}^{p},\;\Delta q_{A}^{r}$), the resulting change of knee joint and hip joint can be calculated as:

$$\Delta q_{K}=arccos\frac{l_{thigh}{}^{2}+l_{tibia}{}^{2}-{\left(l_{A}^{H}-l_{H_{R}}^{H_{L}}*sin\left(\Delta q_{A}^{r}\right)\right)}^{2}}{2l_{thigh}l_{tibia}}$$

(1) $$\Delta q_{H}^{p}=arccos\frac{{\left(l_{A}^{H}-l_{H_{R}}^{H_{L}}*sin\left(\Delta q_{A}^{r}\right)\right)}^{2}+l_{thigh}{}^{2}-l_{tibia}{}^{2}}{2\left(l_{A}^{H}-l_{H_{R}}^{H_{L}}*sin\left(\Delta q_{A}^{r}\right)\right)l_{thigh}}+\Delta q_{A}^{p}$$

**Figure 2:**
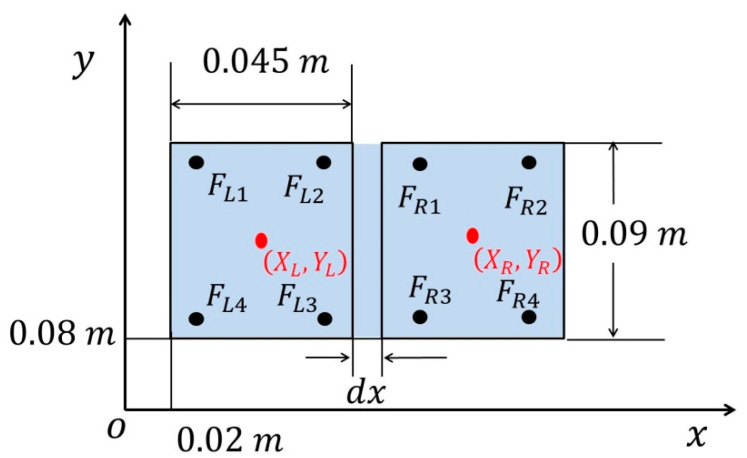
Position of foot sensors and the stable polygon.

Thus, the action space for the proposed RL algorithm is now reduced as $a={\left[\Delta q_{A}^{p},\;\Delta q_{A}^{r}\right]}^{T}$. By combining the IK technique with RL structure, the dimension of action space is significantly reduced, leading to a significantly reduced convergence time. The gyroscope and accelerometers were disabled during the experiments; thus, the only measurable information we were able to obtain was the foot sensor readings. The position of the center of pressure (CoP) in the world frame was defined as two elements of the state space. As shown in Figure 2, the position of CoP is located at the frame, where the support polygon, shown as the blue region, ranges from 0.02 to 0.12 in the x-axis and 0.08 to 0.17 in the y-axis, respectively. Each foot has four foot sensors located at the bottom. The width and length of each foot are 0.045  and 0.09 m, respectively. The distance between the feet in the x direction, dx, is measured in every time step as it may be slightly varying during the experiment.

As can be seen from Figure 2, each foot has four sensors, denoted by F. Thus, the CoP for the left foot along the x-axis and y-axis can be calculated as:

(2) $$X_{CoP}^{L}=\frac{x_{1}F_{L1}+x_{2}F_{L2}+x_{3}F_{L3}+x_{4}F_{L4}}{F_{L1}+F_{L2}+F_{L3}+F_{L4}}$$

(3) $$Y_{CoP}^{L}=\frac{y_{1}F_{L1}+y_{2}F_{L2}+y_{3}F_{L3}+y_{4}F_{L4}}{F_{L1}+F_{L2}+F_{L3}+F_{L4}}$$

where $x_{i}$ and $y_{i}$ denote the x and y coordinates, respectively, for the foot sensors. $F_{\mathrm{Li}}$ is the corresponding sensor readings of the left foot. The CoP of the right foot ($X_{CoP}^{R},\;X_{CoP}^{R}$) can be consequently computed using the same equations. As a result, the position of CoP for the entire robot can be computed as:

(4) $$X_{CoP}=X_{CoP}^{L}\left(\frac{F_{L}}{F_{L}+F_{R}}\right)+\left(X_{CoP}^{R}+d_{x}\right)\left(\frac{F_{R}}{F_{L}+F_{R}}\right)$$

(5) $$Y_{CoP}=Y_{CoP}^{L}\left(\frac{F_{L}}{F_{L}+F_{R}}\right)+Y_{CoP}^{R}\left(\frac{F_{R}}{F_{L}+F_{R}}\right)$$

where $F_{L}$ and $F_{R}$ are the sum of sensor readings of the left and right feet, respectively. The desired CoP is measured $\left(X_{CoP}^{desired}=0.065\;m,\;Y_{CoP}^{desired}=0.116\;m\right)$ while the robot is at its initial position without applying any actions or external disturbances. The state space of the system is now defined as $S={\left[X_{CoP},Y_{CoP},q_{A}^{p},{\dot{q}}_{A}^{p},q_{A}^{r},{\dot{q}}_{A}^{r}\right]}^{T}$, where  q ˙ denotes the angular velocity of the ankle joint in pitch and roll. The goal of the proposed RL algorithm is to make the measured CoP as close to the desired CoP ($X_{CoP}^{desired},\;Y_{CoP}^{desired}$) as possible while the robot is in any state with any disturbance.

## 3. Framework

### 3.1. Framework of the Proposed Hybrid Reinforcement Learning 

Many existing hybrid RL structures attempt to integrate the model-based structure into model-free learning [33,34,35,36]. The model itself is being updated, leading to a faster and more accurate value function estimation, where the sample efficiency is significantly improved. Inspired by their structure, we believe reducing the dimension of action space with a reduced state space will also improve the sample efficiency with a lesser computational load. In addition, according to previous studies, the distribution mismatch problem always exists in their HRL frameworks [33,34,35,36]. This is because these frameworks essentially establish parallel connections between the model-based RL and the model-free RL. Both of the model-based and model-free parts are being updated within the learning loop; thus, two policies are generated parallel from the model-based and model-free parts, respectively. Sometimes, the policy obtained from the predicted model (model-based RL) is completely different from the randomly sampled policy (model-free RL); therefore, further calculations are required to determine which policy is better, leading to less efficiency of the HRL framework. We believe that avoiding the distribution mismatch problem directly is much more straightforward with less computation than finding a method to address it. Thus, alternatively, a novel structure is proposed in this work which establishes a serial connection between the model-based and model-free structures, where the distribution mismatch issue can be avoided. The model-based (MB) estimator is used to estimate the system model and obtain initial control inputs during the offline training procedure. The model-free (MF) optimizer utilizes the control inputs as a reduced action space and fine tunes the action during the learning procedure.

**Figure 3:**
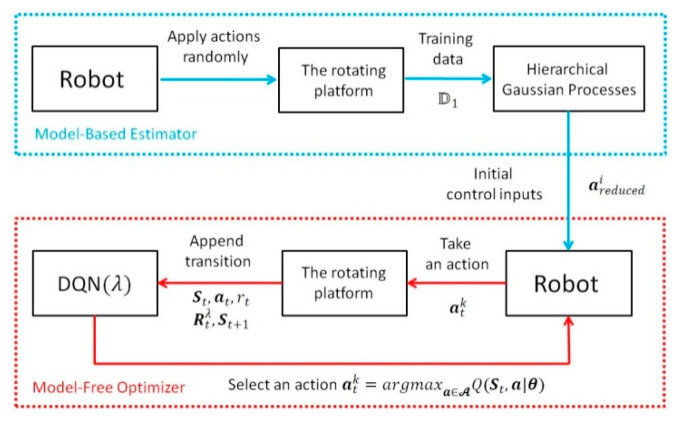
The framework of the proposed hybrid reinforcement learning.

As shown in Figure 3, at the beginning of offline training, the predefined discrete actions are randomly applied to the robot to collect training data $x_{i}$. However, the number of states is still too large for HGP as it requires a huge amount of training data to obtain the estimated model. The computational load will be consequently increased exponentially. Therefore, to reduce the amount of all possible states, we reduced the dimension of the state space by ignoring $X_{CoP},Y_{CoP}$. Thus, the state space for the model-based offline training structure (HGP) was redefined as $s_{HGP}={\left[q_{A}^{p},{\dot{q}}_{A}^{p},q_{A}^{r},{\dot{q}}_{A}^{r}\right]}^{T}$. For simplicity, we use s to represent $s_{\mathrm{HGP}}$ in this section. All training data is stored as an array $D_{1}$ where it contains N training tuples ($x_{i}\in D_{1},\;i=1,2,\cdots ,N$). Each tuple includes three elements that $x_{i}={\left[s_{i},a_{i},s_{i+1}\right]}^{T}$, where $s_{i}$ and $s_{i+1}$ represent the current and the successor states, respectively, and $a_{i}$ in the current random action. 

Then, the HGP was employed to estimate the dynamic model of the system, after which the reduced action space was generated by finding the actions that minimized the immediate cost. The reduced action space here was essentially a four dimensional array, where the first dimension indicated the total state, the second and third dimensions represented the pitch ($\Delta q_{A}^{p}$) and roll *(*$\Delta q_{A}^{r}$) actions, respectively, and the fourth dimension indicated the elements for each action. It is called “reduced” because the control input of each dimension ($\Delta q_{A}^{p}$ and $\Delta q_{A}^{r}$) for each state was not a specific value, but a vector containing several possible actions. This was where the fourth dimension came from. For example, we assumed that the original action space of the change of pitch angle $\Delta q_{A}^{p}$ was defined by K discrete actions ranging from −C to C. Thus, the action space could be written as $\Delta q_{A}^{p}\in \left[-C,C\right]$. The goal of the proposed HGP was to significantly reduce the number of actions for each state, that is, to reduce the range of action space. The HGP could generate a new action space for each state, i.e., the reduced action space, where it only contained k actions ranging from −c to c (k≪K and c≪C). Now, the action space could be written as $\Delta q_{A}^{p}\in \left[-c,c\right]$. 

As a result, the model-free online learning could directly search for the best policy for each state within the reduced action space above. The MBRL structure provided the MFRL with a dramatically reduced action space, leading to a significantly reduced convergence time. Consequently, this novel structure bridged the gap between MBRL and MFRL, which overcame the disadvantages of MFRL by utilizing the advantages of MBRL. Considering the large size of state space, deep Q-learning (DQN) was employed to generate a network to evaluate the value function with respect to all actions. DQN utilized the current measured state $S_{t}$ as the input to generate a hidden layer using feature function ϕ(s); the output were the value functions Q(S,a|θ) weighted by unknown parameters θ. Note that the input state of the network is written in capital S as it includes not only the state for HGP (s) but the position of CoP ($X_{CoP},Y_{CoP}$). The outputs were state–action value functions Q with respect to. all possible actions. A detailed explanation is found in Section 3.4.

### 3.2. Hierarchical Gaussian Processes 

The proposed HGP contained two layers. The first layer generated the initial model of the system w.r.t. each predefined action. The second layer utilized the predicted results from the first layer as an input to generate the final model. We assumed that the action space was defined as $A\in \left[a_{1},a_{2},\ldots a_{N}\right]$, where $a_{i}={\left[\Delta q_{A}^{p}{}_{i},\;\Delta q_{A}^{r}{}_{i}\right]}^{T}$, N is the total number of action pairs. The first layer of HGP was to generate N individual predictions w.r.t. each action pair. The training samples of the first layer could be presented as $D_{1}=\left[D_{1}^{1},D_{1}^{2},\ldots ,D_{1}^{N}\right]$. The training input ($X_{1}=\left[X_{1}^{1},X_{1}^{2},\ldots ,X_{1}^{N}\right]$,$\;X_{1}\in D_{1}$) was the state pairs $X_{1}^{j}={\left\{s_{t}^{j},a^{j}\right\}}_{t\in \left\{1,2,3\ldots ,T\right\}}^{j\in \left\{1,2,\ldots ,N\right\}}$ and the training target ($Y_{1}^{j}\in D_{1}^{j}$) was the successor state $Y_{1}^{j}={\left\{s_{CoP_{t+1}}^{j}\right\}}_{t\in \left\{1,2,3,\ldots ,T\right\}}^{j\in \left\{1,2,\ldots ,N\right\}}$, where j indicates jth action pair from the action space A, $s_{t}^{j}={\left[q_{A}^{p}{}^{j},{\dot{q}}_{A}^{p}{}^{j},q_{A}^{r}{}^{j},{\dot{q}}_{A}^{r}{}^{j}\right]}^{T}$, and $s_{CoP_{\left(t+1\right)}}^{j}={\left[X_{CoP}{}^{j},Y_{CoP}{}^{j}\right]}^{T}$. For simplicity, we use $s_{t+1}$ to represent $s_{\mathrm{CoP}\left(t+1\right)}$ in this section. Given the test input as $X_{1*}^{j}={\left\{s_{i}^{j},a^{j}\right\}}_{i\in \left\{1:M\right\}}^{j\in \left\{1,2,\ldots ,N\right\}}$, the estimated training outputs were the Gaussian distributions of the successor states $s_{CoP_{\left(t+1\right)}}^{j}=E\left[f\left(X_{1*}^{j}\right)|X_{1*}^{j},D_{1}^{j}\right]$, calculated from a multiple outputs GP, corresponding to each action pair  j, shown in Equation (8). L indicates the dimension of output, which, in our case, is 2. $E_{f}$ and ${\mathrm{var}}_{f}$ in Equation (8) are the predicted mean and variance, which can be calculated using Equations (6) and (7), respectively.

(6) $$\mu_{*}≔m_{f}\left(X_{*}\right)=E\left[f\left(X_{*}\right)|X_{*},D\right]=K_{f}\left(X,X_{*}\right){\left(K+\sigma_{n}{}^{2}I\right)}^{-1}y$$

(7) $$\Sigma_{*}≔var_{f}\left[f\left(X_{*}\right)|X_{*},D\right]=K_{f}\left(X_{*},X_{*}\right)-K_{f}\left(X,X_{*}\right){\left(K+\sigma_{n}{}^{2}I\right)}^{-1}K_{f}\left(X_{*},X\right)$$

where $K_{f}$ is the kernel and is defined by the squared exponential function. Then, the second layer multiple outputs GP was employed as the final estimator to predict the final model of the system, where the training input was exactly the predicted output from the first layer. We defined the state space as $s\in \left[s_{1},s_{2},\ldots s_{M}\right]$, where $s_{i}={\left[q_{A}^{p}{}^{i},{\dot{q}}_{A}^{p}{}^{i},q_{A}^{r}{}^{i},{\dot{q}}_{A}^{r}{}^{i}\right]}^{T}\;,\;i\in M$ is the state pair, and M indicates the total number of state spaces. The second layer of HGP was to generate M individual predictions w.r.t. each state pair. The training samples of second layer could be presented as $D_{2}=\left[D_{2}^{1},D_{2}^{2},\ldots ,D_{2}^{M}\right]$. The training input ($X_{2}=\left[X_{2}^{1},X_{2}^{2},\ldots ,X_{2}^{M}\right]$,$\;X_{2}\in D_{2}$) is $X_{2}^{i}={\left\{s^{i},a_{j}^{i}\right\}}_{j\in \left\{1,2,\ldots ,N\right\}}^{i\in \left\{1,2,\ldots ,M\right\}}$ and the training target ($Y_{2}^{i}\in D_{2}^{i}$) is the successor state obtained from the first layer $\;Y_{2}^{i}={\left\{s_{\left(t+1\right)j}^{i}\right\}}_{j\in \left\{1,2,\ldots ,N\right\}}^{i\in \left\{1,2,\ldots ,M\right\}}$. Given the test input as $X_{2*}^{i}={\left\{s^{i},a_{j}^{i}\right\}}_{i\in \left\{1:M\right\}}^{j\in \left\{1,2,\ldots ,N\right\}}$, the estimated training outputs were the Gaussian distributions of the successor states $s_{CoP_{\left(i+1\right)}}^{i}=f\left(X_{2*}^{i}\right)$, calculated from a multiple outputs GP (Equation (8)), corresponding to each state pair  i. Thus, the probability distributions of successor states w.r.t. each state and action pair was obtained.

(8) $$p(s_{t+1}{}^{j}|X_{1},X_{1*},Y_{1})\sim N\left(\left[\begin{array}{c} E_{f}\left[s_{1,t+1}^{j}|X_{1},X_{1*},Y_{1}\right]\\ \vdots \\ E_{f}\left[s_{L,t+1}^{j}|X_{1},X_{1*},Y_{1}\right] \end{array}\right],\left[\begin{array}{ccc} var_{f}\left[s_{1,t+1}^{j}|X_{1},X_{1*},Y_{1}\right]\cdots 0 & \cdots & 0\\ \vdots & \ddots & \vdots \\ 0 & \cdots & var_{f}\left[s_{L,t+1}^{j}|X_{1},X_{1*},Y_{1}\right]\cdots 0 \end{array}\right]\right)$$

The benefit of using hierarchical Gaussian processes was that it significantly reduces the number of training data to fit the model and we only needed to collect the training data once for the first layer of HGP. Then, the estimated model was obtained from the second layer of HGP, where the training data was directly from the first layer without additional training samples. The detailed implementation and explanation of HGP can be found in our previous paper [37].

### 3.3. Initial Control Inputs 

The initial control inputs were calculated by find an action range for each state that minimized the following cost function:

(9) $$E\left[c\left(s_{t+1}\right)\right]=1-{\left|I+\Sigma \Lambda^{-1}\right|}^{-\frac{1}{2\sigma_{e}{}^{2}}}\times exp\left(-\frac{1}{2\sigma_{e}{}^{2}}{\left(s_{t+1}-s_{d}\right)}^{T}\Omega^{-1}\left(s_{t+1}-s_{d}\right)\right)$$

where $\Lambda^{-1}$ is the diagonal precision matrix and the elements in $\Lambda^{-1}$ are unity. $\Omega^{-1}=\Lambda^{-1}{\left(I+\Sigma \Lambda^{-1}\right)}^{-1}$. $s_{d}$ is the desired state. Here we utilize $E\left[c\left(s_{t+1}\right)\right],$ as the expectation of the cost at $s_{t+1}$ is essentially equals to the expectation of the cost at $s_{t}$ after an action $a_{t}$ is applied. Thus, for any given current state $s_{t}$ after taking an action $a_{t}$, the expected immediate cost $E\left[c\left(s_{t+1}\right)\right]$ can be computed using Equation (9), where μ and Σ are the predicted mean and covariance of $p\left(s_{t+1}\right),$ respectively. As a result, the reduced action space is defined as $a_{reduced}^{i}={\left[\Delta q_{A}^{p},\;\Delta q_{A}^{r}\right]}^{T}$ for any state $s_{i},\;i\in M$. Detailed implementation of obtaining the initial control inputs can be found in our previous paper [38].

### 3.4. Model-Free Fine Tuning 

To obtain an accurate policy with minimum variance, the model-free tuning scheme must consider not only the joint information as the state space but also the position of CoP. As a result, the state space here was extended to $S={\left[X_{CoP},Y_{CoP},q_{A}^{p},{\dot{q}}_{A}^{p},q_{A}^{r},{\dot{q}}_{A}^{r}\right]}^{T}$. However, as the number of all possible state pairs was still too large, generating a look-up table (Q-table) and updating it was impractical. Thus, we employed deep Q(λ)-network (DQN (λ)) [39] to find the best policy each state. The traditional Q-learning updates the Q-function $Q\left(s_{t},a_{t}\right)$ at time t+1 after taking an action $a_{t}$; the updating rule can be represented as:

(10) $$Q\left(s_{t},a_{t}\right)=Q\left(s_{t},a_{t}\right)+\alpha \left(r_{t}+\gamma max_{a\in A}Q\left(s_{t+1},a\right)-Q\left(s_{t},a_{t}\right)\right)$$

where $r_{t}$ is the reward at time t, α∈(0,1] is the learning rate, and γ∈(0,1] is the discount factor [40]. But with the increasing number of state pairs, the traditional algorithm was no longer feasible as it required an extremely large look-up table to store the value, where the convergence speed was significantly reduced. DQN replaced the original Q-table with a deep network, where a mapping between the state space and the value function (Q-Function) was generated. Updating the parameterized network was much more time efficient than updating the look-up table. The inputs of the network were the current measured states, and the outputs were the state–action function values. The goal of the model-free fine-tuning algorithm was to find the best action for any given state by updating the parameters. The value of the jth Q-function can be written as:

(11) $$Q\left(s,a_{j}\right)=\overset{n}{\underset{i=1}{\sum }}\phi \left(s_{i}\right)\theta_{i}$$

where $\phi \left(s_{i}\right)$ is the feature, n represents the total number of states. DQN is a successful implementation of deep reinforcement learning (DRL), which resembles a deep neural network and learns a parameterized function $Q\left(s_{t},a_{t}|\theta \right)$ instead to generalize the state pairs. DQN utilizes replay memories D to store transitions $\left(s_{t},a_{t},r_{t},s_{t+1}\right)$ and employs gradient descent to find the parameters θ that minimize the following lose function:

(12) $$ℒ\left(\theta \right)=E_{\left(s_{j},a_{j},r_{j},s_{j+1},\right)\sim D}={\left(r_{j}+\gamma \underset{a\in A}{max}Q\left(s_{j+1},a|\theta^{-}\right)-Q\left(s_{j},a_{j}|\theta \right)\right)}^{2}$$

where the parameter $\theta^{-}$ is a scale copy of θ that fixes the Q-value targets temporarily to prevent divergence or oscillations. On the other hand, the changes of parameters do not impact $\theta^{-}$ immediately; thus, the training inputs do not have to be 100% independent and identically distributed [41]. DQN essentially contains two NN structures, where both $\theta^{-}$ and θ are being updated during the process of learning. With both target network and experience replay, DQN has a more stable input and output to train the network, where the entire scheme of learning behaves like supervised learning. However, one drawback of DQN is that it only uses the current reward to determine the loss function, that is, only the 1-step temporal difference (TD) [40] is used to update the value function:

(13) $$Q(s_{t},a_{t}|\theta )\leftarrow Q\left(s_{t},a_{t}|\theta \right)+\alpha \left(R_{t}^{\left(1\right)}-Q\left(s_{t},a_{t}|\theta \right)\right)$$

As can be seen from Equation (13), the 1-step TD return only utilizes the current $r_{t}$ reward where $R_{t}^{\left(1\right)}=r_{t}+\gamma maxQ\left(s_{t+1},a|\theta \right)$. Only considering the current reward is not enough to generate a better policy as it may introduce overshoot and oscillation in the context of stability control. Thus, we utilized the n-step return to update the value function Equation (13), which can be presented as:

(14) $$R_{t}^{\left(n\right)}=r_{t}+\gamma r_{t+1}+\cdots +\gamma^{n-1}r_{t+n-1}+\gamma^{n}maxQ\left(s_{t+n},a|\theta \right)$$

where 1≤n≤T−n. If n is large, it means that many rewards are considered to evaluate the value function, since the n-step return is equivalent to the Monte Carlo (MC) return, where huge numbers of samples are required for convergence to avoid variance. Sutton provided a tradeoff between the number of rewards and the sampling efficiency, known as the λ-return [39]. The λ-return is defined as the exponential average of all n-step returns:

(15) $$R_{t}^{\lambda }=\left(1-\gamma \right)\overset{T-t-1}{\underset{n=1}{\sum }}\lambda^{n-1}R_{t}^{\left(n\right)}+\lambda^{T-t-1}R_{t}^{T-t}$$

If λ, in Equation (15), equals to 1, the λ-return is the same as the MC algorithm. On the other hand, if λ=0, the λ-return is the same as the 1-step TD algorithm. Thus, it can be treated as a smooth interpolation between TD and MC. The λ-return in Equation (15) is actually the “forward view” [41], which is not directly implementable in practice as it is extremely difficult at each step to predict what will happen many steps later. In addition, it is computationally expensive as it requires n-step trajectory, where $O\left(N^{2}\right)$ operations must be done. It is particularly impractical for a long trajectory. As a result, the “backward view” of λ-return is employed to overcome this drawback, which provides a more efficient updating rule [39]:

(16) $$R_{t}^{\lambda }=r_{t}+\gamma \left(\lambda R_{t+1}^{\lambda }+\left(1-\lambda \right)maxQ\left(s_{t+1},a|\theta \right)\right)$$

Therefore, by applying the “backward” scheme, the computational load is reduced to O(N), which is much more efficient for calculating a long trajectory of returns [40]. In Equation (16), each n-step return becomes a sum of discounted rewards and a final maximization over action values. This is equivalent to Peng’s Q(λ) [42]. Consequently, the integration of λ-return with DQN yields DQN(λ) that allows the λ-return to be updated periodically and stored in the corresponding replay memory. Compared with DQN that stores the current reward $r_{t}$ in replay memory D, the DQN(λ) instead stores several steps of discounted rewards $R_{t}^{\gamma }$ in D, where transitions are appended to a temporary table T. The λ-return is computed along T and stored in D if the state is within the stable region ${\;S}_{s}$. The loss function Equation (12) is now rewritten as:

(17) $$ℒ\left(\theta \right)=E_{\left(s_{j},a_{j},R_{j}^{\gamma }\right)\sim D}{\left(R_{j}^{\lambda }-Q\left(s_{j},a_{j}|\theta \right)\right)}^{2}$$

where

(18) $$R_{j}^{\lambda }=r_{j}+\gamma \left(\lambda R_{j+1}^{\lambda }+\left(1-\lambda \right)Q\left(s_{j+1},a|\theta \right)\right)$$

Note that the parameter $\theta^{-}$ in Equations (17) and (18) is eliminated, compared with Equation (12), as all of the λ-returns are periodically updated using the current Q-function. Thus, it is not necessary to copy θ to $\theta^{-}$ in our case. Instead, all of the λ-return will be updated periodically using the present Q-function, shown in Algorithm 1.

**Algorithm 1** Updating $R_{𝓉}^{\lambda }$1: **For** transition $\left(S_{𝓉},a_{𝓉},r_{𝓉},R_{𝓉}^{\lambda },S_{𝓉+1}\right)$, from back to front **do**2:   **If**
$S_{𝓉+1}$ is terminal3:     Update $R_{𝓉}^{\lambda }\leftarrow r_{𝓉}$4:   **Else**5:     Obtain the next transition from T
$$\left(S_{𝓉+1},a_{𝓉+1},r_{𝓉+1},R_{𝓉+1}^{\lambda },S_{𝓉+2}\right)$$6:     Update $R_{𝓉}^{\lambda }\leftarrow r_{𝓉}+\gamma \left[\lambda R_{𝓉+1}^{\lambda }+\left(1-\lambda \right)max_{a\in A}Q\left(s_{𝓉+1},a\right)\right]$7:   **End If**8: **End For**

This guarantees the fast convergence speed while retaining a stable TD target. For any time step t, the transition will be stored in T first until the current state is within the stable region $S_{s}$. Then, the λ-return for each time step will be calculated from back to front using Algorithm 1. Finally, the temporary table T will be stored in D, after which it will be refreshed as empty. The algorithm will then sample a random minibatch from the replay memory to improve the Q-function, where the parameters θ will be updated using Equation (19).

(19) $$\theta \leftarrow \theta +\alpha \nabla_{\theta }{\left(R_{j}^{\lambda }-Q\left(S_{j},a_{j}|\theta \right)\right)}^{2}$$

Finally, all λ-returns $R_{D}^{\lambda }$ within the replay memory will be calculated by applying Algorithm 1 again. According to the Section 3.3, the reduced action space is computed by Hierarchical GP for all states and can be defined as $a_{reduced}^{i}\in {\mathbb{R}}^{M},\;i=1:M$, where i indicates all possible states belonging to the state space. As illustrated in Section 3.3, $a_{\mathrm{reduced}}^{i}$ is an 4D array containing all possible action pairs for all state pairs ($s_{HGP}={\left[q_{A}^{p},{\dot{q}}_{A}^{p},q_{A}^{r},{\dot{q}}_{A}^{r}\right]}^{T}$), where each state pair corresponds to a 2D vector that contains all possible action pairs for this state. For simplicity, we use $A_{i}$ to represent the reduced action space $a_{reduced}^{i}$. For any censored state $s_{i}$, all possible action pairs at this state can be presented as $A_{i}=\left[a_{i}^{1},a_{i}^{2},\cdots ,a_{i}^{K}\right]$, where K is the total number of action pairs in $A_{i}$, and $a_{i}^{k}={\left[\Delta q_{A}^{p},\;\Delta q_{A}^{r}\right]}^{T}$ is the kth action for state $s_{i}$ and k∈K. DQN (λ), as the model-free optimizer, considers the entire state space that contains the censored joint information as well as the position of CoP. The state space of network is now extended to $S={\left[X_{CoP},Y_{CoP},q_{A}^{p},{\dot{q}}_{A}^{p},q_{A}^{r},{\dot{q}}_{A}^{r}\right]}^{T}$. For any time step, the algorithm calculates the difference between the censored state $S_{t}$ and all state pairs in $s_{HGP}$, and finds the specific $s_{i}$ with minimum difference. Then, the action space $A_{i}$ that corresponds to $s_{i}$ will be selected for DQN (λ) to evaluate the corresponding Q-function values, after which the parameter θ will be updated by applying gradient descent scheme. Detailed implementation of the proposed algorithm is shown in Algorithm 2.

**Algorithm 2** Hybrid Reinforcement Learning Algorithm1: **Initialize**: State space S, and action space A2: Apply discrete actions randomly to the robot and collect data set $D_{1}$3: Using $D_{1}$ to generate $D_{2}$, and then obtain the transition model ℳ4: Obtain the reduced action space $a_{\mathrm{reduced}}^{i}$5: **Initialize**
A using $a_{\mathrm{rough}}^{i}\in \;\left[a_{\mathrm{rough}\left(\min\right)}^{i},a_{\mathrm{rough}\left(\max\right)}^{i}\right]$6: **Initialize** replay memory D, to capacity N, parameter vector θ7: **For**
episode=1,K
**do**8:   Reset the robot and the platform to their initial position, transition temporary table T=∅9:   **For**
t=1, T
**do**10:     Obtain current state $S_{t}$ from sensors’ reading11:     Select a random action from $A_{t}$ with probability ϵ, otherwise select $a_{t}^{k}$
      $a_{t}^{k}={\mathrm{argmax}}_{a\in A}Q(S_{t},a|\theta )$, observe the next state $s_{t+1}$, and receive an immediate reward
$r_{t+1}$
12:     Append transition $\left(S_{t},a_{t},r_{t},R_{t}^{\lambda },S_{t+1}\right)$ to T13:     **If**
$S_{𝓉+1}$ is within stable region $S_{s}$14:       Update $R_{T}^{\lambda }$ using Algorithm 115:       Store T in D, refresh T: T=∅16:     **End If**17:     Sample random minibatch of transitions from $D\left(S_{j},a_{j},r_{j},R_{j}^{\lambda },S_{j+1}\right)$, j=1,2,⋯,P18:     Apply a gradient descent on θ to improve Q-function $\theta \leftarrow \theta +\alpha \nabla_{\theta }{\left(R_{j}^{\lambda }-Q\left(S_{j},a_{j}|\theta \right)\right)}^{2}$19:     Every C step, update $R_{D}^{\lambda }$ using **Algorithm 1**20:    **End For**21: **End For**

## 4. Experiment and Discussion

### 4.1. Experiment Setup 

The experiment environment was built in V-REP. We assumed that all sensor readings contained measurement noises at all times. The total time of offline training procedure for collecting training samples for HGP was 500 episodes, with 20,000 steps for each episode. The training data for the model-based estimator were collected by applying pre-defined discrete actions randomly to the robot, and allowing it to interact with the dynamic platform. The training data were collected as tuples which could be represented by $D_{1}=\left[D_{1}^{1},D_{1}^{2},\ldots ,D_{1}^{121}\right],$ indicating that the total number of action pairs was 121. For each training sample $D_{1}^{j}$, the training input and training target could be represented as $X_{1}^{j}={\left\{s_{t}^{j},a^{j}\right\}}_{t\in \left\{1:T\right\}}^{j\in \left\{1,2,\ldots ,121\right\}}$ and $Y_{1}^{j}={\left\{s_{CoP_{t+1}}^{j}\right\}}_{t\in \left\{1:T\right\}}^{j\in \left\{1,2,\ldots ,121\right\}}$, respectively, where j and T are the action index and the total training time, respectively. The probability distribution of successor states weas obtained using the first layer of HGP. The training input is given by $X_{1*}^{j}={\left\{s_{i}^{j},a^{j}\right\}}_{i\in \left\{1:14641\right\}}^{j\in \left\{1,2,\ldots ,121\right\}}$, and the probability distributions of the successor states $E\left[f\left(X_{1*}^{j}\right)|X_{1*}^{j},D_{1}^{j}\right]$ can be analytically computed. The results were the estimated distributions of the successor states specified by 121 individual transition functions. For the second layer GP, the training data were the estimated outputs from the first layer. The training data for the second layer can be defined as $D_{2}^{i}={\left\{s_{i}^{i},a_{k}^{i},s_{i+1}^{i}\right\}}_{k\in \left\{1,2,\ldots ,121\right\}}^{i\in \left\{1,2,\ldots ,14641\right\}}$, where there were 14,641 state pairs in total. The output was 14,641 individual transition functions, indicating the estimated distributions of the successor states $E\left[f\left(X_{2*}^{i}\right)|X_{2*}^{i},D_{2}^{i}\right]$. As mentioned in Section 3.2, the cost is defined as the geometric distance between the predicted state, i.e., the predicted CoP: $\left(x_{p},y_{p}\right)$ and the desired CoP $\left(x_{d}=0.065\;m,\;y_{d}=0.116\;m\right)$, which can be written as:

(20) $$c=1-exp\left(-\frac{1}{2\sigma_{e}{}^{2}}\sqrt{{\left(y_{p}-y_{d}\right)}^{2}+{\left(x_{p}-x_{d}\right)}^{2}}\right)$$

Therefore, the reduced action space could be obtained by minimizing the expected immediate cost using Equation (9). Then, DQN(λ) was employed as the model-free fine-tuning scheme to find the best action pair for each state. The total training episode was 322 with 20,000 steps for each episode. Instead of allowing the simulation to run forever, the episodic tasks were considered in our work. At the beginning of each episode, the robot and the platform were reset to their initial positions, and the temporary table T was initialized to empty. This is because the entire experiment, including the offline training process and the online learning procedure, assumed that the robot frame (local frame) was translated from the world frame without rotation, which meant that the x-axis, y-axis, and z-axis of the robot frame were pointing the same directions as the world frame. However, during the experiment, some of the intense actions produced large instant velocity of the robot joints, leading the robot to deviate from the original local frame. The resulting robot frame may have rotated from the world frame, which consequently introduced errors after applying the control inputs. Episodic tasks allowed the robot to be able to recalibrate the consistency between the robot frame and the world frame, which avoided the error and increased the efficiency of the algorithm. The total number of online learning episodes was 2200. The reward function is defined as:

$$r=\{\begin{array}{cc} -10 & Falling\;Down\\ 10 & Stable\;Region\\ exp\left(-\frac{1}{2}{\left(S-S_{d}\right)}^{T}\Psi^{-1}\left(S-S_{d}\right)\right) & Otherwise \end{array}$$

where Ψ is the diagonal weighting matrix with the elements of 0.5, where each element determines the impact of the corresponding state. Considering that the final goal of the algorithm was to maintain the robot balance where the CoP must be located within the desired region, the joint angles had to vary from each state as the angle of the platform was changing; thus, joint angles were not considered as part of the desired state. Furthermore, to reduce the oscillation, the angular velocities had to be as small as possible. As a result, the stable state only contained the position of CoP as well as the angular velocities of the ankle joint, which can be presented as $S_{d}={\left[0.065,0.116\right]}^{T}$. The stable region is defined as ${\left[0.065\pm 0.01,\;0.116\pm 0.005\right]}^{T}$.

### 4.2. Experiment Results 

After obtaining the reduced action space using the proposed HGP, the robot and the platform were reset to their initial positions, after which the online learning scheme was conducted.

**Figure 4:**
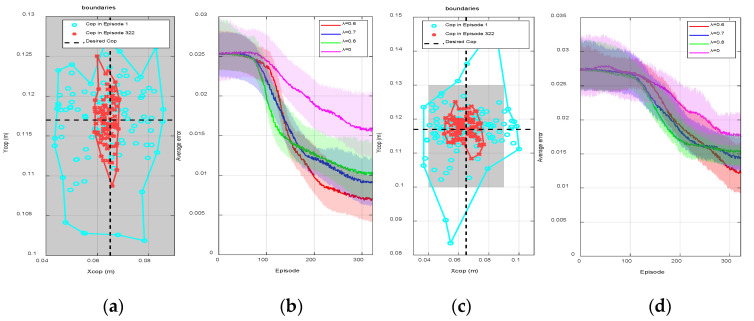
(**a**) One hundred random sensor readings and boundaries at episodes 1 and 322 for a single trail when λ=0.6, $f_{y}=f_{x}=0.0333\;\mathrm{Hz}$, (**b**) the average episode error from 40 individual trails with respect to learning episode for different λ ($f_{y}=f_{x}=0.0333\;\mathrm{Hz}$ ), (**c**) 100 random sensor readings and boundaries at episodes 1 and 322 for a single trail when λ=0.6, $f_{y}=f_{x}=0.0833\;\mathrm{Hz}$, (**d**) the average episode error from 40 individual trails with respect to learning episode for different $\lambda \cdot f_{y}=f_{x}=0.0833\;\mathrm{Hz}$.

**Figure 5:**
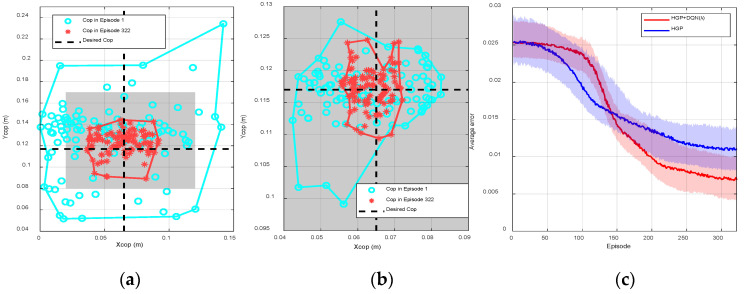
(**a**) One hundred random sensor readings and boundaries at episodes 1 and 322 using only deep Q-learning (DQN(λ)) without hierarchical Gaussian processes (HGP) for a single trail when λ=0.6, $f_{y}=f_{x}=0.0333\;\mathrm{Hz}$, (**b**) 100 random sensor readings and boundaries at episodes 1 and 322 using only HGP when $f_{y}=f_{x}=0.0333\;\mathrm{Hz}$, (**c**) the average episode errors from 40 individual trails using the proposed hybrid reinforcement learning (RL) and only using the HGP, respectively.

The goal of implementing the proposed model-free online learning algorithm to NAO robot on a rotating platform was to obtain a controller that is able to control the robot and maintain its balance on the rotating platform with different frequencies and different maximum magnitudes at all times. As can be seen from Figure 4 and Figure 5, the sampling points (CoP) of episode 1, indicated by blue circles, are located within the predefined support region at the beginning. This meant that HGP had effectively provided an initial control strategy, where the robot was able to maintain balance but with a relatively large CoP error. Consequently, this rough control strategy led to a reduced action space which enabled the MF optimizer to find the optimal within a small range of actions. Both figures show the procedure of online learning where the platform is rotating along the x-axis and y-axis at the same time. The maximum rotating magnitudes of the platform along x-axis and y-axis were both 20 deg. The frequency of the platform along x-axis $\left(f_{x}\right)$ and y-axis $\left(f_{y}\right)$ were 0.033 and 0.0833 Hz, respectively. 

As shown in Figure 4a, we collected 100 random sensor readings from the beginning of learning (episode 1) to the end of learning (episode 322) from a single trail to show the process of convergence. The gray area represents the stable region of the biped robot. The blue circles and red cross markers are the real time measured position of CoP. The black dash lines are the desired CoP in the y-axis and x-axis, respectively. The more the sample points are close to the intersection of two dash lines, the more the robot is stable. The algorithm is trying to make the CoP of the robot as close to the intersection as possible. 

At the beginning of learning, the sampling points were scattered ranging from 0.04 to 0.09 m in the x-axis and 0.1 to 0.13 m in the y-axis. The boundaries, represented by the blue curve in Figure 4a, in the x-axis and y-axis were (0.045 m, 0.086 m) and (0.103 m, 0.126 m), respectively. During the process of learning, the sampling points tended to concentrate at the desired steady state, the average error was consequently reduced. At the end of learning (episode 322), the sampling points were scattered ranging from 0.058 to 0.085 m in the x-axis and 0.1055 to 0.125 m in the y-axis. The boundaries, indicated by the red curve, in the x-axis and y-axis were reduced to (0.058 m,0.068 m) and (0.108 m,0.125 m), respectively. Figure 4b shows the average episode errors from 40 individual trials with respect to learning episodes when λ is different, where the error for each episode is calculated by averaging the values from 40 randomly initialized trials at the same episode. As can be seen from episode 90 to episode 150, the convergence speed was relatively faster if λ was larger (λ=0.8). However, small λ (λ=0.6) led to small steady state error and thus the control performance was better. Compared with DQN (λ=0), DQN(λ) showed better overall performance, where the convergence speed was increased and the steady state error was reduced. The trained controller was consequently defined as the obtained actions for all states after the learning process when λ=0.6. The robustness of this controller will be further tested in the next several experiments. 

Then, we increased the rotating frequency of the platform to 0.0833 Hz to imitate the increase of the external disturbances, where the system was initialized and the policy was re-trained from the beginning. The trained policy can maintain the balance of the robot that all sampling points are within the support polygon; however, compared with Figure 4a, the collected sampling points are more dispersed meaning that the steady state performance is worse than before. As can be seen from Figure 4c, at the beginning of the training process, the boundaries are increased to (0.038 m,0.1 m) and (0.083 m, 0.147 m) for the x-axis and y-axis, respectively. During the process of learning, the error in Figure 4c was always larger than that in Figure 4a. At the end of learning, the boundaries were increased to (0.05 m, 0.078 m) and (0.108 m, 0.126 m) in the x-axis and y-axis, respectively. The average errors shown in Figure 4d were also increased for all λ, compared with those in Figure 4b. As a conclusion, training the policy on the rotating platform with the minor disturbance was easier than with intense disturbance; the training outcome was highly dependent on the platform frequency.

Figure 5a shows the CoP positions and boundaries at episode 1 and episode 322 without applying HGP. Compared with Figure 4a, the robot was unstable at many time steps as the corresponding positions of CoP were outside of the stable region. Although the robot was eventually stable at episode 322, the steady state errors, ±0.03 and ±0.025 m  in the x-axis and y-axis, respectively, were still much larger than the results in Figure 4a. Figure 5b shows the CoP positions and boundaries before and after the learning process using HGP without DQN(λ). At the beginning, as indicated by the blue circles in Figure 4a and Figure 5b, there is almost no difference between using HGP only and using HGP+ DQN(λ). However, after the learning process, the CoP boundary of using HGP ((0.055 m,0.073 m) in the x-axis and (0.1059 m, 0.125 m) in y-axis) was larger than using HGP+ DQN(λ) ((0.058 m,0.068 m) in the x-axis and (0.108 m,0.125 m) in y-axis), which are presented by red star markers in Figure 4a and Figure 5b, respectively. Figure 5c also indicates the average error during the learning procedure. The red curve is the average errors using HGP as the estimator and DQN(λ) as the optimizer, which is exactly the same red curve in Figure 4b. The blue curve shows the convergence of only using HGP. The convergence speed was faster at the beginning, but it gradually reduced after episode 140. The steady state error was eventually larger than using DQN(λ), since the model contained measurement noises leading to an inaccurate control input to the robot. As a result, Figure 5a proves the effectiveness of using HGP as the estimator and Figure 5b,c indicates the effectiveness of using DQN(λ) as the optimizer. The proposed hybrid RL structure was able to improve the sample efficiency and reduce the steady state error.

We compared our results (Figure 4 and Figure 5) with Wu’s [21] results, whose work also focused on the stability control of a biped robot on a rotating platform. The average CoP error of our work was 0.0125 m, while the error of their paper was approximately 0.025 m on average. In addition, our model was more complex (2 DoF of the platform) than that in their study (only 1 DoF), which meant it was much more difficult and challenging for the controller to balance the robot since more disturbances were introduced in our work. Detailed comparisons are listed in Table 1 below.

As a result, it made sense that to obtain a controller that can better control the robot to maintain stability, the training procedure had to be done on a platform with minor rotating frequencies, where both control performance and convergence speed could be guaranteed. Further experiments were consequently being conducted using the trained controller obtained from Figure 4 under the condition of λ=0.6, $f_{y}=f_{x}=0.0333\;\mathrm{Hz}$. More complex environments were introduced in the following experiments by increasing $f_{x}$ during the experiment. The robustness of the trained controller was tested under 6 different fixed rotating frequencies. We extracted 100 sampling points from each of these experiments to show the stability of the robot, as well as the control performance of the controller.

**Figure 6:**
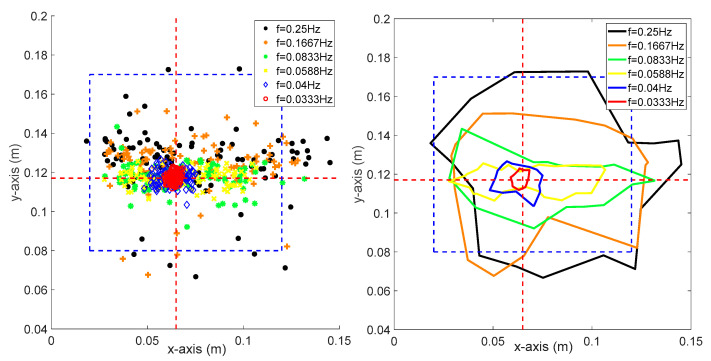
One hundred sampling points and the corresponding boundaries where $f_{x}$ is ranging from 0.0333 to 0.25 Hz.

As can be seen from Figure 6, the blue region indicates the support polygon of the robot, where the robot is stable if the sampling point is within this area but unstable if the point is outside this region. The red circles indicate the location of CoP, where the robot was controlled by the trained controller (same as Figure 4, episode 2200). As the frequency increased, the error between the desired stable state with the measured state was increased accordingly. The figure on the right side indicates the boundaries of CoP of different frequencies. The robot fell down if the frequency of the platform was larger than 0.0588 Hz, indicated by the yellow line; thus, the success rate of the policy was not 100% anymore. We further tested the robustness as well as the control performance of the controller under a more complicated environment by increasing $f_{x}$ and $f_{y}$ at the same time. 

**Figure 7:**
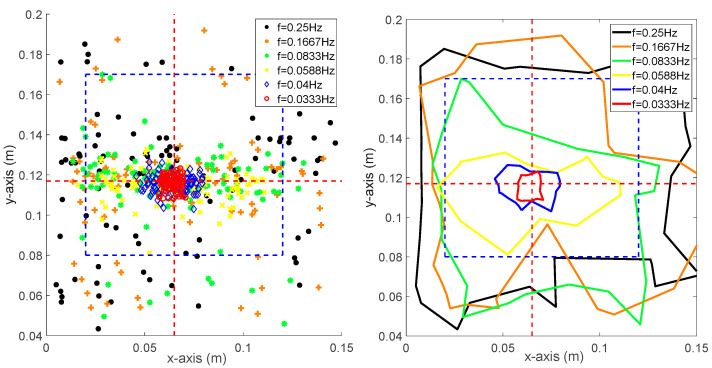
One hundred sampling points and the corresponding boundaries where $f_{x}$ and $f_{y}$ are both ranging from 0.0333 to 0.25 Hz.

As shown in Figure 7, the figure on the left side shows the sampling points of CoP under different platform frequencies, the right figure indicates the boundaries of these points. Compared with Figure 6, it can be easily seen that the sampling points are more scattered from the desired stable state and the corresponding boundaries are extended. Therefore, it can be concluded that the robot tends to be more unstable if $f_{x}$ and $f_{y}$ are larger. According to Figure 6 and Figure 7, the resulting success rate is highly dependent on the frequency of the platform. If the frequency was large, it meant that the external disturbance was relatively intense; thus, the robot was not able to maintain balance perfectly.

Both of the pitch and roll frequencies affected the overall performance of the robot. To explore the relation between the success rate and the platform frequencies, we utilized the trained controller (obtained via the experiment shown in Figure 4) to test how both frequencies affected the success rate. Figure 9 shows the area of success rate ranging from 60% to 100% with respect to different frequencies.

**Figure 8:**
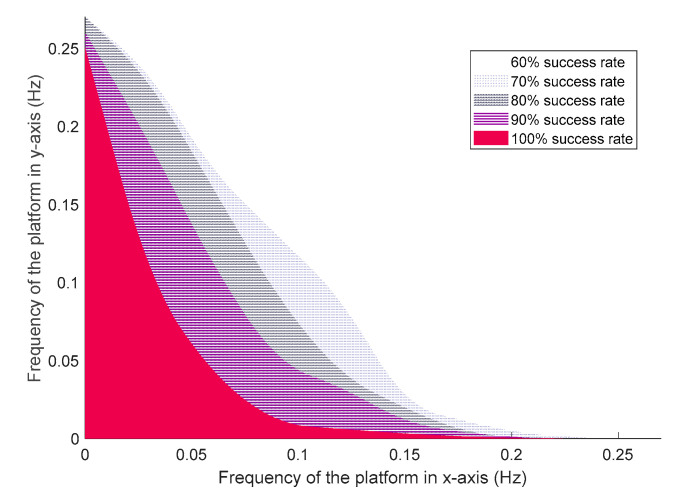
The bandwidth of biped stability on the rotating platform with respect to different success rates.

As shown in Figure 8, $f_{y}$ and $f_{x}$ both range from 0 to 0.27 Hz. The red area indicates the bandwidth of the platform that allows the robot to maintain balance with a 100% success rate (no falling detected). The maximum $f_{y}$ was 0.25 Hz, while the maximum $f_{x}$ was 0.21 Hz in this experiment. With the increase of $f_{x}$, from 0 to 0.1 Hz, the $f_{y}$ was decreased significantly from 0.25 to 0.009 Hz, after which it gradually reduced to zero. The maximum $f_{y}$ of 90% success rate was increased to 0.26 Hz, while the maximum $f_{x}$ was still 0.21 Hz. With the reduced success rate, the maximum $f_{y}$ was maintained at 0.27 Hz. For 70% and 60% success rates, the maximum $f_{x}$ was increased to 0.23 and 0.26 Hz, respectively. As a conclusion, the system was more sensitive to $f_{x}$ than $f_{y}$, since the action at the knee joints and hip joints were calculated using the predefined constraints equations, a subtle change of roll angle of ankle joins led to a significant change in other joints, and the resulting CoP would consequently change dramatically.

During the experiment, we found that most fallings were detected while the platform was at its maximum rotating magnitude, the robot was more stable if the magnitude was relatively small. Thus, we believe that the performance of the controller was also dependent on the magnitude of the platform, where the robot was more likely to fall if the magnitude was large, and the success rate was consequently reduced. As a result, we conducted two experiments individually to show how magnitude influenced the success rate with different frequencies. For simplicity, the maximum magnitude of pitch and roll are represented by $m_{y}$ and $m_{x}$, respectively. The maximum magnitude in one direction was fixed while the other was changing during each experiment. Only 100% success rate cases were considered.

**Figure 9:**
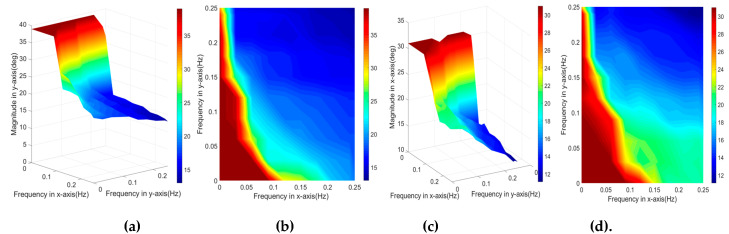
(**a**) A 3D view of maximum pitch magnitude of the platform with 100% success rate for different frequencies, (**b**) 2D view of maximum pitch magnitude, (**c**) 3D view of maximum roll magnitude of the platform with 100% success rate for different frequencies, (**d**) 2D view of maximum roll magnitude.

As shown in Figure 9, with the increased platform frequencies, both $m_{y}$ and $m_{x}$ are reduced. To guarantee the 100% success rate, the maximum magnitude was sensitive to the change of frequencies, particularly when $f_{y}$ ranged from 0.009 to 0.25 Hz, while $f_{x}$ ranged from 0 to 0.1 Hz.

Figure 9 indicates the change of $m_{y}$ while $f_{x}$ and $f_{y}$ are changing simultaneously. It reaches 38 deg if both frequencies are small, represented by the red area. Then it drops drastically to 15 deg with the increasing frequencies, after which $m_{y}$ reduces smoothly to 13 deg. The same result for $m_{x}$ can be found in Figure 9c,d, where $m_{x}$ equals to 32 deg for small frequencies and it is also sensitive for moderate frequencies. The difference between $m_{y}$ and $m_{x}$ is that $m_{x}$ was more sensitive for high frequencies and, indicated by blue area, it continued dropping significantly and reduced to 10.5 deg at an end. The reason why the magnitude could induce great influence on the system is that with the increased magnitude, the angle of the platform was also increased, where the support polygon projected on the flat ground was decreased. According to the principle of ZMP [4], for static biped robots, CoP had to be within the support polygon so that the robot could be stable. However, it was extremely hard for the controller to apply actions at the ankle joints to adjust the Centre of Gravity (CoG) and make the CoP located within the reduced region. A subtle change of joint angle could have induced a drastic change of CoG, where the resulting CoP would be changed significantly.

## 5. Conclusions

In this paper, a hybrid reinforcement learning framework consisting of the model-based scheme and the model-free framework was proposed and applied to a NAO robot to maintain balance on a dynamic platform. By adding the inverse kinematics, the dimension of action space was reduced before offline training. Then, a hierarchical Gaussian processes containing two layers was employed as the MB estimator to estimate the transition model between the robot and the environment, after which the initial control inputs, i.e., a reduced action space, were obtained for online tuning. Finally, the model-free online learning method-DQN (λ) was applied as the model-free optimizer to evaluate the reduced action space, and find the best action for each state. Compared with other GP-based model-based RL algorithms that utilize state action pairs to predict the transition model, the proposed hierarchical GP employed two layers of prediction that estimated the transition model twice, where it enabled the algorithm to estimate the system model with few training samples, and consequently increased the training time. Moreover, after obtaining the rough model of the system, the model-based estimator was no longer being updated during the online learning procedure. As a result, the computational load for online learning was reduced. The sample efficiency for online learning was improved as the model-free optimizer only needed to evaluate a small number of possible actions for each state. The proposed hybrid reinforcement learning structure also avoided the distribution mismatch problem when integrating model-free RL into model-based RL. Simulation results showed that the proposed HRL algorithm guaranteed the efficiency of model-based RL while it still achieved the steady state performance of model-free RL. The algorithm was able to control NAO to maintain balance on a rotating platform with different frequencies and magnitudes, where the robustness of the controller was also guaranteed. Future works will involve the combination between the inverse kinematics and RL as well as the extension of the state and action space to generate the walking gait for NAO robots to imitate human walking patterns. The algorithm will also be improved and be further applied on walking gait generation to enable the biped robot to be able to walk on a flat surface, inclined or declined surfaces, and on a dynamic platform. Further experiments for biped walking control will involve the comparisons between the improved HRL and other algorithms, such as soft actor critic, asynchronous advantage actor critic, and deep deterministic policy gradient. The algorithm will also be implemented into a physical robot to test the performance of the learning algorithm in the real environment with real external disturbances.

## Figures and Tables

**Table 1:** Results comparisons between the proposed HRL method and Wu’s method [21].

Evaluation Index	Our Method	Wu’s Method
ZMP Error	0.0125 m	0.025 m
DoF of the platform	2	1
Maximum angle	±38 deg	±30 deg
Maximum frequency	0.0333 Hz	0.25 Hz
Success rate (0.0333 Hz, ±30 deg)	100%	82%
